# Identification of Differentially Expressed Genes in Different Glioblastoma Regions and Their Association with Cancer Stem Cell Development and Temozolomide Response

**DOI:** 10.3390/jpm11111047

**Published:** 2021-10-20

**Authors:** Justin Bo-Kai Hsu, Tzong-Yi Lee, Sho-Jen Cheng, Gilbert Aaron Lee, Yung-Chieh Chen, Nguyen Quoc Khanh Le, Shiu-Wen Huang, Duen-Pang Kuo, Yi-Tien Li, Tzu-Hao Chang, Cheng-Yu Chen

**Affiliations:** 1 Department of Medical Research, Taipei Medical University Hospital, Taipei 110, Taiwan; justin.bokai@gmail.com (J.B.-K.H.); 154101@h.tmu.edu.tw (G.A.L.); shiuwen@tmu.edu.tw (S.-W.H.); 2 Translational Imaging Research Center, Taipei Medical University Hospital, Taipei 110, Taiwan; 161003@h.tmu.edu.tw (S.-J.C.); s19501062@gm.ym.edu.tw (Y.-C.C.); khanhlee@tmu.edu.tw (N.Q.K.L.); 194029@h.tmu.edu.tw (D.-P.K.); angela810727@tmu.edu.tw (Y.-T.L.); 3 Warshel Institute for Computational Biology, The Chinese University of Hong Kong, Shenzhen 518172, China; leetzongyi@cuhk.edu.cn; 4 School of Life and Health Science, The Chinese University of Hong Kong, Shenzhen 518172, China; 5 Department of Medical Imaging, Taipei Medical University Hospital, Taipei 110, Taiwan; 6 Department of Microbiology and Immunology, School of Medicine, College of Medicine, Taipei Medical University, Taipei 110, Taiwan; 7 Professional Master’s Program in Artificial Intelligence in Medicine, College of Medicine, Taipei Medical University, Taipei 110, Taiwan; 8 Research Center for Artificial Intelligence in Medicine, Taipei Medical University, Taipei 110, Taiwan; 9 Department of Pharmacology, School of Medicine, College of Medicine, Taipei Medical University, Taipei 110, Taiwan; 10 Graduate Institute of Medical Sciences, College of Medicine, Taipei Medical University, Taipei 110, Taiwan; 11 Neuroscience Research Center, Taipei Medical University, Taipei 110, Taiwan; 12 Graduate Institute of Biomedical Informatics, Taipei Medical University, Taipei 110, Taiwan; 13 Clinical Big Data Research Center, Taipei Medical University Hospital, Taipei 110, Taiwan; 14 Department of Radiology, School of Medicine, College of Medicine, Taipei Medical University, Taipei 110, Taiwan

**Keywords:** glioblastoma, tumor recurrence, TMZ resistance, biomarker

## Abstract

The molecular heterogeneity of gene expression profiles of glioblastoma multiforme (GBM) are the most important prognostic factors for tumor recurrence and drug resistance. Thus, the aim of this study was to identify potential target genes related to temozolomide (TMZ) resistance and GBM recurrence. The genomic data of patients with GBM from The Cancer Genome Atlas (TCGA; 154 primary and 13 recurrent tumors) and a local cohort (29 primary and 4 recurrent tumors), samples from different tumor regions from a local cohort (29 tumor and 25 peritumoral regions), and Gene Expression Omnibus data (GSE84465, single-cell RNA sequencing; 3589 cells) were included in this study. Critical gene signatures were identified based an analysis of differentially expressed genes (DEGs). DEGs were further used to evaluate gene enrichment levels among primary and recurrent GBMs and different tumor regions through gene set enrichment analysis. Protein–protein interactions (PPIs) were incorporated into gene regulatory networks to identify the affected metabolic pathways. The enrichment levels of 135 genes were identified in the peritumoral regions as being risk signatures for tumor recurrence. Fourteen genes (*DVL1*, *PRKACB*, *ARRB1*, *APC*, *MAPK9*, *CAMK2A*, *PRKCB*, *CACNA1A*, *ERBB4*, *RASGRF1*, *NF1*, *RPS6KA2*, *MAPK8IP2*, and *PPM1A*) derived from the PPI network of 135 genes were upregulated and involved in the regulation of cancer stem cell (CSC) development and relevant signaling pathways (Notch, Hedgehog, Wnt, and MAPK). The single-cell data analysis results indicated that 14 key genes were mainly expressed in oligodendrocyte progenitor cells, which could produce a CSC niche in the peritumoral region. The enrichment levels of 336 genes were identified as biomarkers for evaluating TMZ resistance in the solid tumor region. Eleven genes (*ARID5A*, *CDC42EP3*, *CDKN1A*, *FLT3*, *JUNB*, *MAP2K3*, *MYBPC2*, *RGS14*, *RNASEK*, *TBC1D30*, and *TXNDC11*) derived from the PPI network of 336 genes were upregulated and may be associated with a high risk of TMZ resistance; these genes were identified in both the TCGA and local cohorts. Furthermore, the expression patterns of *ARID5A*, *CDKN1A*, and *MAP2K3* were identical to the gene signatures of TMZ-resistant cell lines. The identified enrichment levels of the two gene sets expressed in tumor and peritumoral regions are potentially helpful for evaluating TMZ resistance in GBM. Moreover, these key genes could be used as biomarkers, potentially providing new molecular strategies for GBM treatment.

## 1. Introduction

Glioblastoma multiforme (GBM) is the most common malignant primary brain tumor in adults [1]. Despite the continual development of relevant treatment strategies and standards of care, including maximal safe resection, chemotherapy, and radiotherapy, the current median survival time far is still only approximately 15 months [2,3,4]. Additionally, tumor relapse and drug resistance are inevitable outcomes for patients with GBM. Tumor heterogeneity, including at the molecular and cellular levels, can be the cause of such poor prognoses [5]. Therefore, determining the links between tumor heterogeneity, GBM recurrence, and drug resistance could facilitate the development of effective treatment regimens against GBM.

Certain molecular characteristics are correlated with GBM recurrence, namely the promoter methylation and expression of the O-6-methylguanine-DNA methyltransferase (*MGMT*) gene, as well as various mutations and RNA expression levels of other genes [6]. The methylated promoter of *MGMT* has been associated with a favorable prognosis for patients with GBM [7,8,9,10]. *MGMT* overexpression results in GBM recurrence; however, *MGMT* expression is independent of promoter methylation [11]. Some genes related DNA mismatch repair and cell stemness have been identified as gaining mutations [12] and being differentially expressed [13] between primary and recurrent GBM. These characteristics suggest that recurrent GBM can develop and be regulated due to multiple factors, some of which may affect treatment responses [14].

Temozolomide (TMZ) is the most commonly used chemotherapy drug for patients with GBM. This drug can easily penetrate the blood–brain barrier and is effective in treating gliomas, potentially prolonging the median overall survival of affected patients [15]. By alkylating a tumor’s genomic DNA, TMZ can induce GBM cell death by inducing nucleotide mismatch and boosting the mismatch repair pathway [16]. However, during the course of treatment, GBM develops TMZ resistance. The main molecular characteristics are correlated with DNA repair pathways, including *MGMT* upregulation and the upregulation of genes involved in base excision repair. These genes can repair TMZ-induced lesions and preserve a tumor’s genomic integrity. Many other factors are linked to TMZ resistance, such as the presence of the wild types of isocitrate dehydrogenase 1 (*IDH1*) and telomerase reverse transcriptase (*TERT*) genes and the overexpression of *IDH1*, *TP53*, *EGFR*, and *ATRX* [17].

Despite the fact that multiple molecular features of GBM have been identified in different studies, it is difficult using these features to evaluate the degrees of tumor developing in recurrence and TMZ resistance for patients with primary GBM. In other words, following the favorable degree of tumor development, patients would be capably divided into high and low risk groups. Moreover, most of such features have been identified from data sets covering bulk tumors but not various tumor regions (e.g., tumor mass and tumor margin). Relevant molecular characteristics require investigation to improve GBM treatment strategies and the prognosis of affected patients. Therefore, we sought to identify potential biomarkers of tumor progression in various regions of primary GBM that could aid tumor therapy.

## 2. Materials and Methods

### 2.1. Study Cohorts

Human gene expression data were obtained from public databases, namely The Cancer Genome Atlas (TCGA, https://www.cancer.gov/tcga, accessed on 1 September 2021) and the Gene Expression Omnibus (GEO) [18], and from the medical records of the local study cohort. Data related to different tumor types were from TCGA (154 primary and 13 recurrent tumors) and the local cohort (29 primary and 4 recurrent tumors). Additionally, data from the local cohort related to different tumor regions (29 tumor and 25 peritumoral regions) were obtained using a magnetic resonance imaging (MRI)-guided approach. Moreover, RNASeq (level 1) data from TCGA were downloaded using the Genomic Data Commons (GDC) data transfer tool; we were authorized by the Electronic Research Administration (eRA) Commons and the Database of Genotypes and Phenotypes (dbGaP) to access these level 1 data. A different data platform, namely Agilent microarray, was used for samples collected from the local cohort. When the quality of an RNA library from a sample did not meet experimental requirements, the region-specific samples from recruited patients were excluded. Furthermore, single-cell RNA sequencing (scRNASeq) data for four patients with primary GBM from GEO (GSE84465) were used in this study. This study analyzed 3589 cells extracted from various tumor regions (2343 in the tumor region and 1246 in the peritumoral region), and the cell types included astrocytes, immune cells, neoplastic cells, neurons, oligodendrocytes, and oligodendrocyte progenitor cells (OPCs) had been identified in previous study [19]. The study has been reviewed and approved by the Joint Institutional Review Board of Taipei Medical University (code: N201901041).

### 2.2. The Workflows of the Study

**Figure 1:**
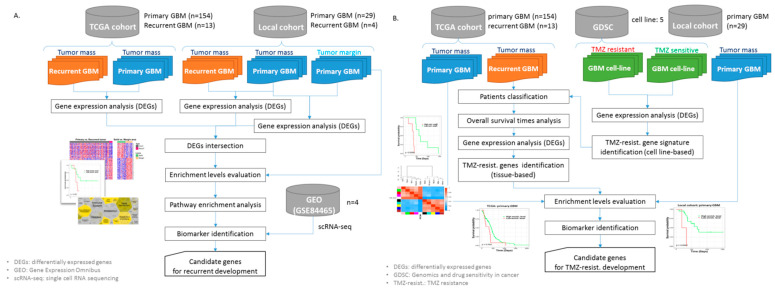
The analysis workflows in the study. (**A**) The workflow of identification of genes related to development of recurrent GBM. (**B**) The workflow of identification of genes related to development of TMZ resistance.

In this study, different analysis workflows were used to identify substantial critical biomarkers regarding development of tumor recurrence and TMZ resistance in different tumor regions of GBM (as shown in Figure 1). In this study, the sample sizes of healthy controls were limited (TCGA cohort, *n =* 5; local cohort, *n =* 1), therefore, we did not do the comparison with healthy tissue.

### 2.3. Genomic Data Preprocessing

The next-generation data (RNASeq/scRNASeq) used in this study were processed in several steps. Two major processes were employed, namely reads alignment (GRCh38 assembly) and the estimation of quantitative gene/isoform expression. For these two processes, we employed HISAT2 [20] and StringTie [21,22], respectively. The transcripts per millions (TPM) method [23] was employed to determine gene expression levels. The formula for TPM is as follows:

(1) $$\mathrm{TPM}=10^{6}\times \frac{reads\;mapped\;to\;transcript/transcript\;length}{Sum\left(reads\;mapped\;to\;transcript/transcript\;length\right)}$$

The GBM dataset on the Agilent microarray platform and the intensities of genes were normalized using the R function “normalize.quantiles”, which was helpful for comparing gene expression levels among samples.

### 2.4. Differentially Expressed Gene Analysis

Because of the limited sample size of specific groups used in the study comparisons, the gene expression of each subsample did not have a normal distribution. Thus, differentially expressed genes (DEGs) between two samples were examined using a Wilcoxon rank-sum test, which was implemented using the R function “*wilcox.test*”. The log2-fold change was obtained from the log base 2 of the ratio of median expression values based on a comparison of sample groups. The statistical significance of DEGs was represented by *p* values obtained from statistical tests.

### 2.5. Gene Set Enrichment Analysis

The conditions of patients with GBM may vary in terms of tumor development, prognosis, and drug response owing to the properties of different tumors and their pathway preferences. Thus, gene signatures from previously identified DEGs could represent the pathway preferences of tumors if the enrichment levels of those genes were to be quantified using Gene Set Enrichment Analysis (GSEA) [24,25]. The enriched levels of specific gene signatures were evaluated using the normalized enrichment score (NES). Moreover, samples with different levels of gene set enrichment were identified through prognosis analysis using the R function “*survMisc*”.

### 2.6. Identification of GBM Recurrence-Associated Gene Signatures

Relevant DEGs were identified and compared between patients with recurrent and primary GBM. Some of these DEGs may have been involved in specific pathways leading to the development of tumor cells in primary tumor margins. Thus, other DEGs of peritumoral and tumor regions of primary GBM were further compared with DEGs related to tumor recurrence. Functional categories of common DEGs were then analyzed using WGCNA [26]. Moreover, the potential regulatory network of these genes was constructed based on protein–protein interactions (PPIs), data for which were downloaded from the BIOGRID database [27], to determine pathway involvement. To analyze the pathway involvement of genes of interest, two pathway-related databases, namely Reactome [28] and KEGG [29], and a pathway-related R function “*pathview*” were used in the study [30]. Therefore, common DEGs could potentially be used as gene signatures within margins of primary tumors and linked to tumor development.

### 2.7. Identification of Tissue-Based Gene Signatures of GBM Sample Responses to TMZ

Generally, the clinical annotations of downloaded tumor sample data did not have drug response information, such as TMZ resistance and sensitivity. Thus, the TMZ response-associated gene signatures within samples could not be evaluated. However, several tumor cell lines have previously been used as standard models for these distinct drug responses. The TMZ response-associated gene signatures, which could be identified in cell line-based gene expression data, were helpful in distinguishing TMZ-resistant tumor samples from among samples from patients with recurrent GBM.

#### 2.7.1. Cell Line-Based TMZ-Resistant Gene Signatures

The gene expression microarray data of T98G, LN-18, U-87-MG, U251, and A172 GBM cell lines were downloaded from the GDSC database [31]. T98G and LN-18 cell lines were used for studying TMZ resistance, and U-87-MG, U251, and A172 were used to study TMZ sensitivity [32]. This enabled DEGs to be identified in the gene expression profiles of TMZ-resistant and TMZ-sensitive cell lines. A *p* value of <0.01 was used to indicate significant DEGs, and such DEGs were filtered and identified as candidate genes related to TMZ response. Subsequently, the expression profiles of all genes within TMZ resistance-related cell lines were collapsed using the “*collapseRows*” function of the R package “*WGCNA*” [26], with the parameter method set as “MaxMean”. Additionally, z-score transformation of the collapsed expression profiles was conducted. Finally, the z-scores of the candidate genes were used as TMZ resistance-associated gene signatures.

#### 2.7.2. Tumor Tissue-Based TMZ-Resistant Gene Signatures

Gene expression profiles of GBM recurrence samples (*n* = 13) from TCGA were compared with the aforementioned gene signatures of cell lines with TMZ resistance. Spearman’s correlation coefficient (*r*) was used to evaluate the degree of TMZ resistance of recurrent tumors, and the expression profiles of tumors with relatively high similarity to gene signatures were suggested to indicate higher resistance to TMZ treatment. Additionally, the survival times of patients were used to identify the optimal r value cutoffs, and “*survMisc*” was used to differentiate recurrent tumors into two groups with different TMZ resistance levels. Subsequently, tumor tissue-based TMZ response-associated gene signatures were identified using DEG analysis.

## 3. Results

### 3.1. Patient Characteristics

Three data cohorts of GBM were used in this study; the demographic characteristics of patients are shown in Table 1. Gene sets potentially related to tumor recurrence were identified in samples of primary (*n =* 183) and recurrent (*n =* 17) GBM from TCGA and local cohort data. Region-associated samples from local cohort data (*n =* 29) and GEO data (*n =* 4) were then further used to identify region-specific biomarkers of tumor recurrence. Potential drug response-associated gene sets were identified in samples with tumor recurrence (*n =* 13) from TCGA. Similarly, the region-specific biomarkers of drug resistance were identified in primary tumor samples from TCGA (*n =* 154) and local cohort data (*n =* 29).

### 3.2. GBM Recurrence-Associated Gene Signatures in Tumor Margins

**Figure 2:**
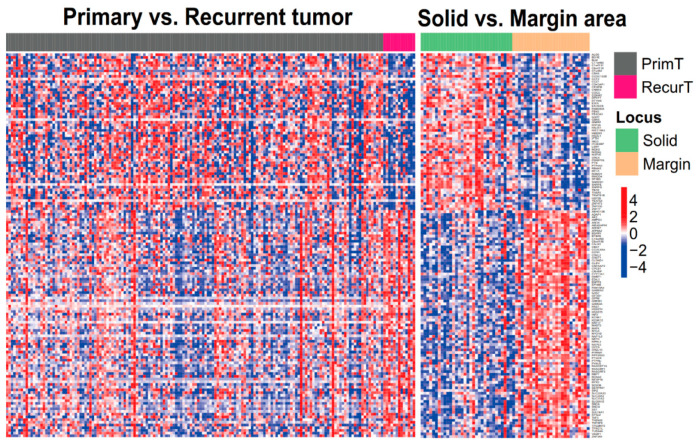
GBM recurrence-associated genes in primary and recurrent tumors. The expression levels of 135 genes relevant to GBM tumor recurrence were similar to the expression levels of genes in the tumor margin of patients with primary GBM. Upregulated genes are in red, and downregulated genes are in blue. Samples and genes are presented on the x and y axes, respectively. Samples from different tumor types and regions are shown using various color bars.

**Figure 3:**
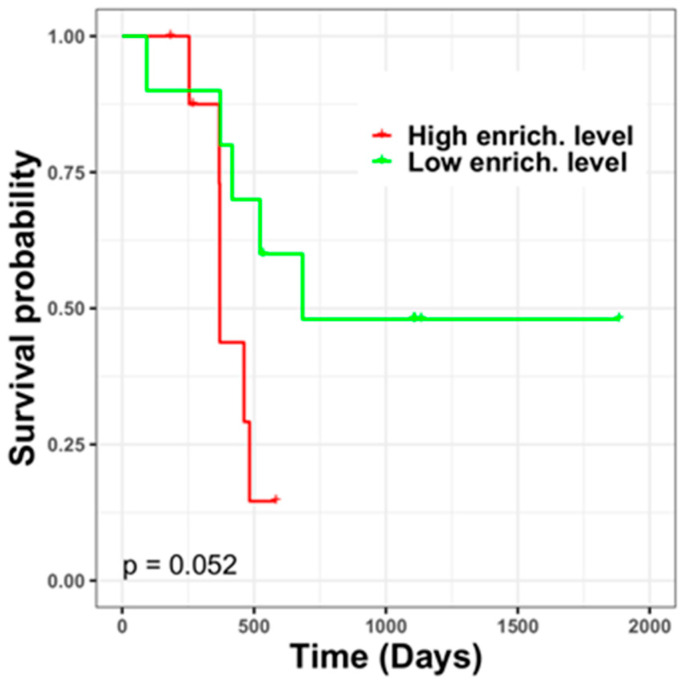
Prognosis of patients with different enrichment levels of GBM recurrence-associated genes within the tumor margin. Patients could be divided based on prognosis; the group (*n =* 9) with a high level of gene enrichment had a poor prognosis, and the group (*n =* 10) with a low level of gene enrichment had a favorable prognosis.

In total, 173 recurrence-associated genes were commonly expressed in recurrent and primary GBM samples from TCGA and our local cohort. Moreover, 135 of such genes were further identified in tumor margin and tumor mass regions of patients with primary GBM, including 78 upregulated and 57 downregulated genes in the tumor margin; the genes were also revealed in recurrent tumor that compared with primary tumor (as shown in Figure 2) these genes are listed in Table S1. Such genes might fundamentally give rise to a relapse niche in the GBM tumor margin. Thus, the enrichment levels of these genes were further used to evaluate the survival duration and risk of recurrence among patients with primary GBM (*n =* 19) who had corresponding tumor margin samples. Following an analysis of gene signatures in the tumor margin, patients were divided into two groups with different prognoses (Figure 3). The high-risk group (*n =* 9) had enriched levels of related genes relative to the low-risk group (*n =* 10), and the difference in the survival curves of the two groups tended toward statistical significance according to a rank-sum test.

### 3.3. Pathway Involvement of GBM Recurrence-Associated Gene Signatures and Potential Biomarkers of Their Regulatory Network

**Figure 4:**
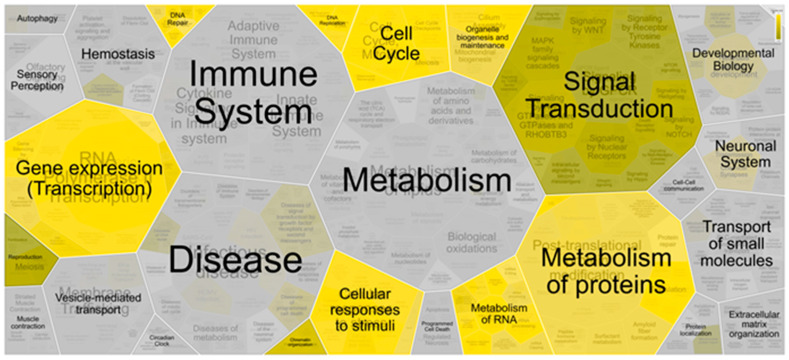
Pathway involvement of critical genes in the regulatory network. Tumor recurrence genes were involved in pathways related to various activities; these genes had the highest involvement in signal transduction and protein metabolism. When genes are matched and significantly overrepresented in a pathway (*p* < 0.05), the pathway is indicated in a yellow to dark yellow gradient (*p* is from 0 to 0.05).

To further elucidate the biological roles of the identified tumor recurrence-associated genes, a putative regulatory network was constructed based on PPIs. All genes in the network had significant differences in expression between tumor margin and tumor mass and between high- and low-risk groups with various degrees of recurrence. After filtering, 864 genes remained within the regulatory network, which were potentially related to recurrence; of these, 788 were derived from PPI relationships and 76 genes were from the identified recurrence-associated gene signatures. Moreover, the pathway involvement of these 864 genes was analyzed using the Reactome tool; the results are shown as the hierarchical Voronoi visualization (Figure 4). Many different pathways were correlated with these genes; however, several pathways involved in signal transduction, including the Notch, Hedgehog, WNT, and MAPK signaling pathways, were related to cancer stem cell (CSC) development, as indicated in several studies [33,34,35]. Fourteen genes (*DVL1*, *PRKACB*, *ARRB1*, *APC*, *MAPK9*, *CAMK2A*, *PRKCB*, *CACNA1A*, *ERBB4*, *RASGRF1*, *NF1*, *RPS6KA2*, *MAPK8IP2*, and *PPM1A*) were further identified within these signaling pathways and were highly expressed in the poor prognosis group, as shown in Figures S1–S4 and Table 2. Ten of the fourteen genes (*DVL1*, *PRKACB*, *APC*, *MAPK9*, *CAMK2A*, *CACNA1A*, *ERBB4*, *NF1*, *MAPK8IP2*, and *PPM1A*) were mainly expressed by oligodendrocyte progenitor cells (OPCs) located in the tumor margin, as determined through single-cell sequencing data analysis (Table 3). Moreover, a study indicated that OPCs could cooperate with macrophages or microglia to form a CSC niche at the tumor border [36].

### 3.4. GBM Cell Line-Based TMZ Resistance-Associated Gene Signatures

**Figure 5:**
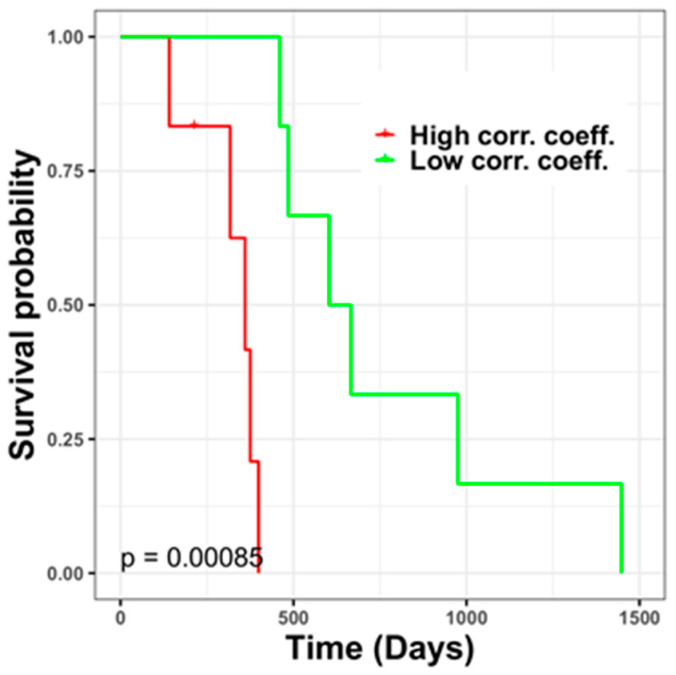
Prognosis of patients whose gene signatures have different degrees of correlation with TMZ resistance-associated genes. Patients whose gene expression profiles had a higher similarity to gene signatures of TMZ-resistant cell lines had a shorter survival time (*n =* 6, poor prognosis and indicated by a red line) than patients with a lower profile similarity. Patients (*n =* 6) with favorable prognosis indicated by a green line.

On the basis of a gene expression analysis of GBM cell lines, 199 genes were correlated with TMZ responses. Of these, 176 genes are commonly used in different gene expression platforms, such as RNASeq and microarray. Thus, a reference of TMZ resistance-associated gene signatures was constructed using the TMZ-resistant cell lines based on these genes (Table S2). Subsequently, the gene expression profiles of patients with recurrent GBM (*n =* 13) were compared with the gene signatures, and degrees (high/low) of similarity were estimated using correlation coefficients. Patients whose gene expression profiles had a high similarity with the indicated gene signatures were considered to have a higher risk of having TMZ-resistant GBM than patients whose profiles had a low similarity with the aforementioned gene signatures. Patients were divided into high- and low-risk groups (*n =* 6 each) with significant differences in prognosis, and the median Spearman correlation coefficient (*r* = 0.32) was used as the cutoff value (Figure 5).

### 3.5. GBM Tissue-Based TMZ Resistance-Associated Gene Signatures

**Figure 6:**
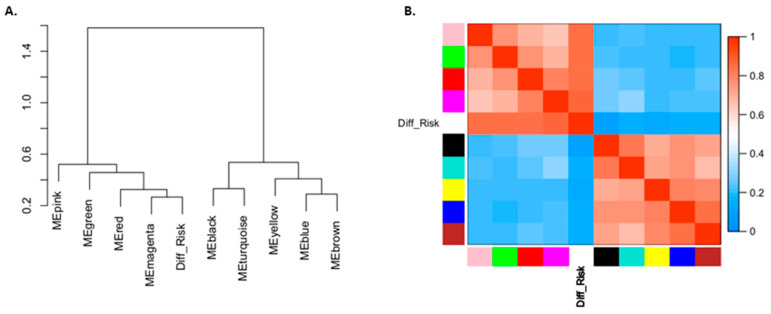
Visualization of the relationships between gene modules and risk of TMZ resistance. (**A**) In the dendrogram, pink, green, red, and magenta modules of genes were significantly correlated with TMZ resistance in recurrent tumor samples. (**B**) The heatmap further demonstrates that these four modules could be clustered together with samples having distinct TMZ resistance risks.

Because recurrent GBMs with high and low risks of TMZ resistance were revealed, 1484 tissue-based DEGs that could be associated with TMZ response were identified. A further analysis of these genes using WGCNA revealed that 350 of them (from four modules) were related to different degrees of TMZ resistance (Figure 6), including 14 upregulated genes and 336 downregulated genes in high-risk patients with recurrent GBM; these genes are listed in Table S3. Moreover, the biological roles of these genes were analyzed using Gene Ontology enrichment analysis, and the biological process of genes in gene module (MEmagenta) which was the most closely related to the different degrees of TMZ resistance could be correlated to various nucleotides transportation between cells; the results are listed in Table S4.

### 3.6. Different TMZ Resistance Risks and Potentially Related Biomarkers within the Tumor Mass of Primary GBM

**Figure 7:**
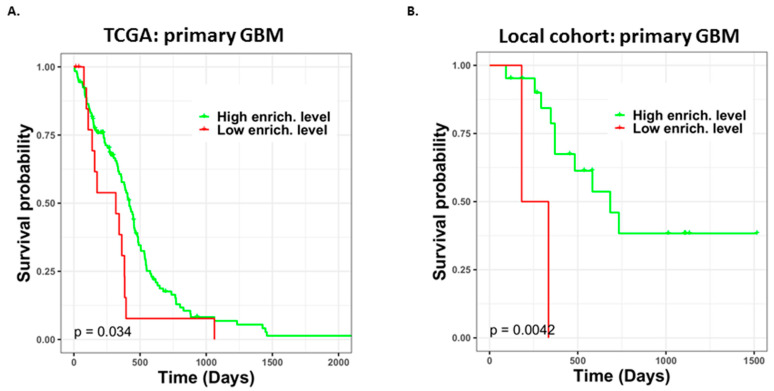
Prognosis evaluation of primary GBM patients with different enrichment levels of TMZ resistance-associated genes. Patients with high enrichment of such genes had favorable prognosis, and patients with a low enrichment of such genes had poor prognosis. (**A**) Different enrichment levels (low level, *n =* 15; high level, *n =* 126) of patients with primary GBM from TCGA cohort. (**B**) Different enrichment levels (low level, *n =* 2; high level, *n =* 21) of patients with primary GBM from the local cohort.

Most primary GBM tumors respond to TMZ treatment; nevertheless, we sought to identify features related to TMZ responses in patients with primary GBM. Therefore, we used the identified TMZ resistance-associated gene set in a GSEA to evaluate the enrichment levels of such genes and their association with patient prognosis. In the analysis, we only focused on the *IDH* wild type. In total, 336 genes were used to divide patients into two risk groups; this was done using TCGA (*n =* 141) and local cohort (*n =* 23) data, as shown in Figure 7 The aforementioned genes were upregulated in patients with GBM recurrence who were identified as potentially having a low risk of TMZ resistance in this study, suggesting that the resistance risk increases at higher enrichment levels. By assessing both TCGA and local cohorts, we determined that in patients (TCGA cohort, *n =* 15 and 2 for TCGA and local cohorts, respectively) with poor prognosis, the presence of genes with low enrichment levels was correlated with TMZ resistance. A putative regulatory network of 336 genes was then constructed based on their relationships with PPIs. In total, 6332 genes were involved in the network; however, only 35 genes were significantly differentially expressed between the two risk groups in terms of the tumor mass of patients with primary GBM. The 11 upregulated genes (*ARID5A*, *CDC42EP3*, *CDKN1A*, *FLT3*, *JUNB*, *MAP2K3*, *MYBPC2*, *RGS14*, *RNASEK*, *TBC1D30*, and *TXNDC11*) in the high-risk group were recognized as putative biomarkers of TMZ resistance in the tumor mass of patients with primary GBM.

## 4. Discussion

In this study, different gene signatures were identified from the tumor margin and tumor mass that could be used to distinguish patients with a high risk (poor prognosis) of developing recurrent tumor and TMZ resistance within primary GBM. Moreover, potential biomarkers of CSC development and TMZ resistance were further filtered through a regulatory network of the identified gene signatures. Compared with the low-risk group, the high-risk group had significantly higher expression levels of associated genes.

A recent study identified seeds for recurrence in the tumor edge; when the tumor core was exposed to treatment-related pressure, edge cells had an increased capacity to promote infiltrative growth, malignancy, and therapy resistance through paracrine crosstalk with the tumor core [37]. The enrichment levels of the 135 identified genes were used to divide patients into two groups with different survival durations, and such genes were possibly active in a gene regulatory network to drive certain pathway reactions causing recurrence at the tumor resection site. Thus, it is reasonable to suggest that these 135 genes play a role in regulating recurrence at the tumor margin.

Constructing a comprehensive regulatory network based on the 135 genes made it possible to determine which pathways were involved in tumor recurrence. In Figure 3, 10 categories of biological pathways related to recurrence are highlighted, namely DNA repair, DNA replication, the cell cycle, organelle biogenesis and maintenance, signal transduction, gene expression (transcription), reproduction, chromatin organization, RNA metabolism, and protein metabolism. Studies have indicated that certain cellular pathways related to tyrosine kinase receptor activation are correlated with GBM recurrence; however, treatments involving the various inhibitors of these receptors have limitations [38,39]. Moreover, pathways relevant to CSC development were suggested as a worthy research target [39]. Therefore, we focused on CSC-associated pathways, such as the Notch, Hedgehog, WNT, and MAPK signaling pathways, and identified 14 genes that could be used as potential biomarkers. Further analysis using single-cell sequencing data demonstrated that most of 14 genes were mainly expressed by OPCs and parts of such genes were expressed by immune cells, indicating that these genes had a role in the formation of a CSC cell niche [36]. However, from our data, we could not determine whether immune cells were macrophages or microglia. Therefore, further investigation of these immune cells is necessary.

Regarding TMZ resistance in GBM treatment, several conclusions in related studies have been based on GBM cell lines with previously understood responses to TMZ (resistance or sensitivity) [32,40]. However, those results were limited to real situations in which tumor tissue was able to resist TMZ treatment through the actions of multiple pathways [41]. In most patients, their recurrent GBM is resistant to TMZ treatment, which is the first-line chemotherapeutic agent for this disease [42]. This may indicate that recurrent tumors have various degrees of TMZ resistance. Therefore, the present study used several GBM cell lines with various TMZ responses to identify response-associated gene signatures. Subsequently, on the basis of these gene signatures, patients with recurrent GBM were divided into two groups with different survival times, representing high and low risks of TMZ resistance.

To our knowledge, the present study is first to use cell line-based TMZ resistance-associated gene signatures to identify recurrent GBM patients with different TMZ resistance risks. We further analyzed the relationships of gene expression profiles and different risk groups to reveal tissue-based TMZ-resistant gene signatures (350 genes). Finally, we attempted to determine the relationships of the enrichment levels of those gene signatures in the tumor mass of primary GBM with patient survival times. Only the enrichment levels of 336 genes were related to the survival times of patients with primary GBM and capable to distinguish the relative high risk of TMZ resistance; these genes were originally identified in recurrent GBM patients with a low risk of TMZ resistance and were upregulation. This is because most patients with primary GBM had an initial response to TMZ treatment, whereas only fewer ones possibly had a poor treatment response. In addition, primary GBM patients were divided into two groups based on prognosis. A regulatory network of these 336 TMZ-associated genes was constructed, and we filtered out genes with expression levels that differed between risk groups of patients with primary GBM. Eleven common upregulated genes in high-risk groups among different data cohorts were recognized as potential biomarkers of TMZ resistance in primary GBM. Some were suggested to be involved in relevant pathways that could affect TMZ responses [41]. For example, *ARID5A* deficiency could decrease ROS generation, thus modulating autophagy [43]. The biological function of *CDC42EP3* was correlated with DNA damage repair [44], and *CDKN1A* could be involved in the AKT pathway’s mediation of the TMZ resistance of glioma cells [45]. *FLT3* is a class III receptor tyrosine kinase that can amplify *EGFR*, resulting in PI3K/Akt pathway dysregulation [41]. The transcription factor *JUNB* plays a crucial role in TMZ resistance, especially in upregulating DNA repair and cancer stemness genes [46]. The other potential biomarkers might be novel ones involved in specific pathways related to TMZ responses.

## 5. Conclusions

The outcome of current GBM treatment strategies remains limited, especially in patients with tumor recurrence. Many researchers have attempted to develop multimodal and combination therapies, none of which have improved patient prognosis. The present study identified distinct gene signatures of GBM recurrence and TMZ resistance in the different tumor regions of primary GBM, potentially facilitating patient selection for personalized treatments. Furthermore, the identified biomarkers of GBM recurrence and TMZ resistance provide a new avenue for targeted GBM therapy.

## Figures and Tables

**Table 1:** Demographic characteristics of patients with GBM in this study.

Study Cohort		TCGA Cohort		Local Cohort		GSE84465
Tumor Type		Primary (*n =* 154)	Recurrent (*n =* 13)	Primary (*n =* 29)	Recurrent (*n =* 4)	Primary (*n =* 4)
*IDH*	WT	139	12	21	0	3
	Mutant	10	1	0	0	0
	n/a	5	0	8	4	1
Age	Median (Q1–Q3)	60.0 (52.0–70.0)	58.0 (48.0–63.0)	62.0 (54.0–70.0)	53.5 (45.8–59.0)	n/a
Gender	Male	99	8	19	2	0
	Female	54	5	10	2	0
	n/a	1	0	0	0	4
Survival (Days)	Median (Q1–Q3)	342.5 (153.0–515.5)	399.0 (317.0–603.0)	413.0 (286.2–696.2)	396.5 (294.2–624.2)	n/a

**Table 2:** Expression fold change of GBM recurrence-associated biomarkers between regions and risk groups.

	Tumor Margin Relative to Tumor Mass (Diff. Regions)		High Risk Relative to Low Risk (in the Tumor Margin)	
	Log2FC	*p*	Log2FC	*p*
*DVL1*	0.199	0.048	1.147	0.000
*PRKACB*	0.930	0.000	1.434	0.000
*ARRB1*	1.145	0.001	1.155	0.017
*APC*	0.711	0.003	1.046	0.006
*MAPK9*	0.495	0.001	0.863	0.008
*CAMK2A*	2.875	0.042	5.115	0.008
*PRKCB*	2.710	0.001	2.749	0.002
*CACNA1A*	1.074	0.030	1.811	0.000
*ERBB4*	1.181	0.005	0.875	0.017
*RASGRF1*	1.694	0.000	1.915	0.000
*NF1*	0.132	0.045	0.690	0.003
*RPS6KA2*	1.123	0.000	0.744	0.009
*MAPK8IP2*	0.746	0.007	1.533	0.004
*PPM1A*	0.464	0.005	0.741	0.000

Log2: ratio of median gene expression values; Log2FC: log2-fold change. *p*: *p* values evaluated using a Wilcoxon rank-sum test analyzing two populations.

**Table 3:** CSC development-associated genes were mainly expressed by OPCs at the tumor border.

	Astrocyte	Immune Cell	Neoplastic	Neuron	Oligodendrocyte	OPC	Vascular	Total Expr. Cells
*DVL1*	9	21	7	7	7	**44**	n/a	95
*ARRB1*	20	**61**	2	4	1	7	n/a	95
*PRKACB*	35	76	29	19	31	**280**	1	471
*APC*	76	66	34	17	19	**199**	n/a	411
*MAPK9*	14	60	12	11	7	**71**	1	176
*CAMK2A*	3	1	n/a	14	n/a	**21**	n/a	39
*PRKCB*	n/a	**109**	1	15	4	96	n/a	225
*CACNA1A*	2	153	3	7	1	**161**	n/a	327
*ERBB4*	44	1	15	19	8	**166**	n/a	253
*RASGRF1*	n/a	6	2	**16**	2	4	n/a	30
*NF1*	48	148	19	15	15	**181**	2	428
*RPS6KA2*	27	**92**	3	17	11	84	1	235
*MAPK8IP2*	11	2	2	9	n/a	**61**	n/a	85
*PPM1A*	16	52	11	8	9	**110**	1	207

Total expr. cells: genes are expressed by total cell counts. Numbers in bold indicate that the relevant gene was mainly expressed by the corresponding cells.

## Data Availability

The datasets analyzed in this study are available from the Genomic Data Commons (GDC) Data Portal of National Cancer Institute (https://portal.gdc.cancer.gov, accessed on 1 September 2021).

## Supplementary Materials

The following materials are available online at https://www.mdpi.com/article/10.3390/jpm11111047/s1, Figure S1: Potential recurrence-associated biomarkers in the Notch signaling pathway. Figure S2: Potential recurrence-associated biomarkers in the Hedgehog signaling pathway. Figure S3: Potential recurrence-associated biomarkers in the WNT signaling pathway. Figure S4: Potential recurrence-associated biomarkers in the MAPK signaling pathway. Table S1: Identified recurrence-associated genes. Table S2: TMZ resistance-associated gene signatures (cell-line based). Table S3: Identified TMZ resistance-associated genes. Table S4: Functional category of TMZ response-associated gene modules.
