# Supplementary material for: Identification of Differentially Expressed Genes in Different Glioblastoma Regions and Their Association with Cancer Stem Cell Development and Temozolomide Response
Source: J Pers Med. 2021 Oct 20;11(11):1047. doi: 10.3390/jpm11111047 (PMC8625522; doi:10.3390/jpm11111047)
Supplement: Supplementary file 1 [file jpm-11-01047-s001.zip › Supplementary_figures.pdf]

Journal of Personalized Medicine

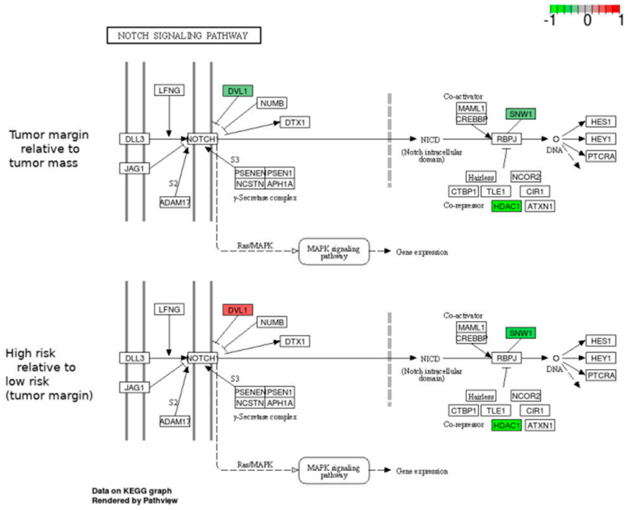

Figure S1. Potential recurrent-associated biomarkers in NOTCH signaling pathway. DVL1 was identified in this signaling pathway.

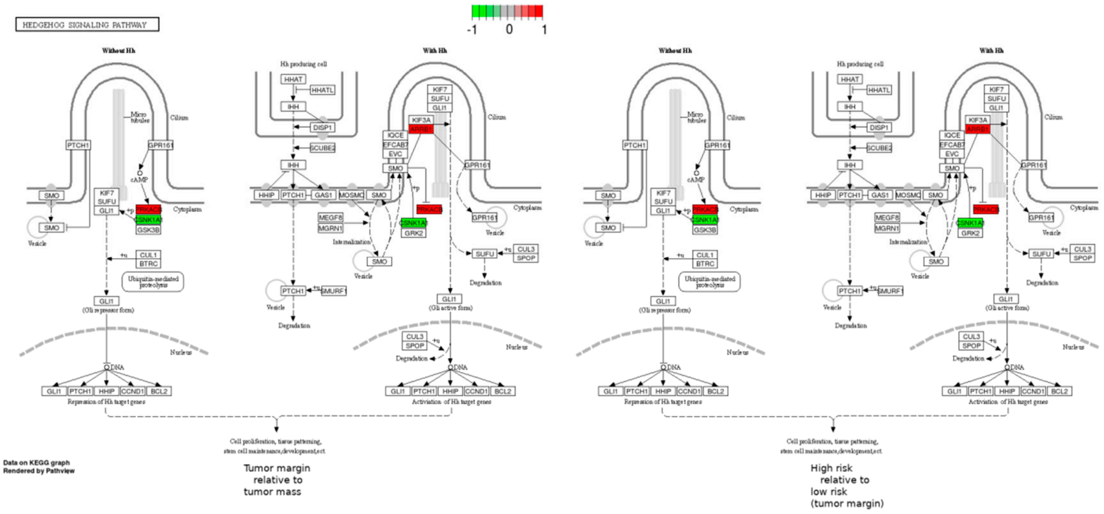

Figure S2. Potential recurrent-associated biomarkers in HEDGEHOG signaling pathway. PRKACB and ARRB1 were identified in this signaling pathway.

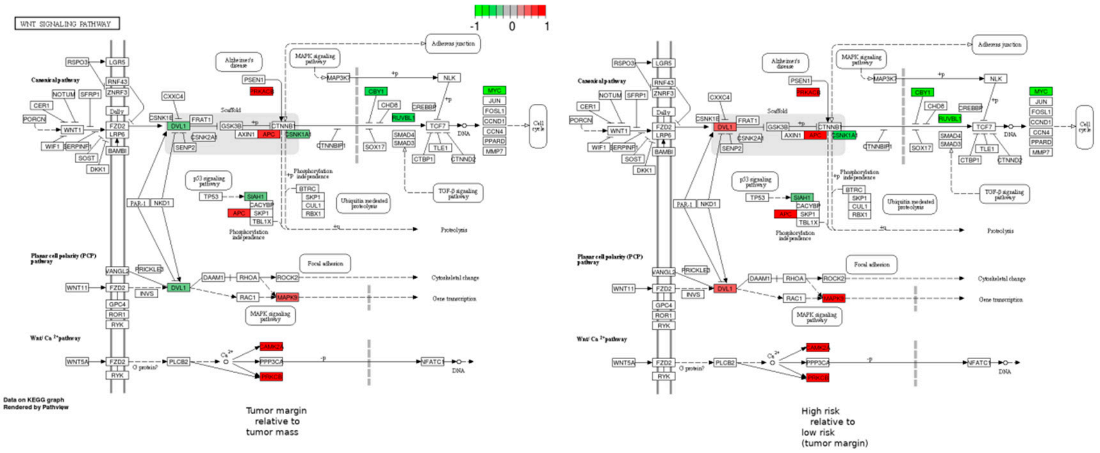

Figure S3. Potential recurrent-associated biomarkers in WNT signaling pathway. PRKACB, DVL1, APC, MAPK9, and CAMK2A were identified in this signaling pathway.

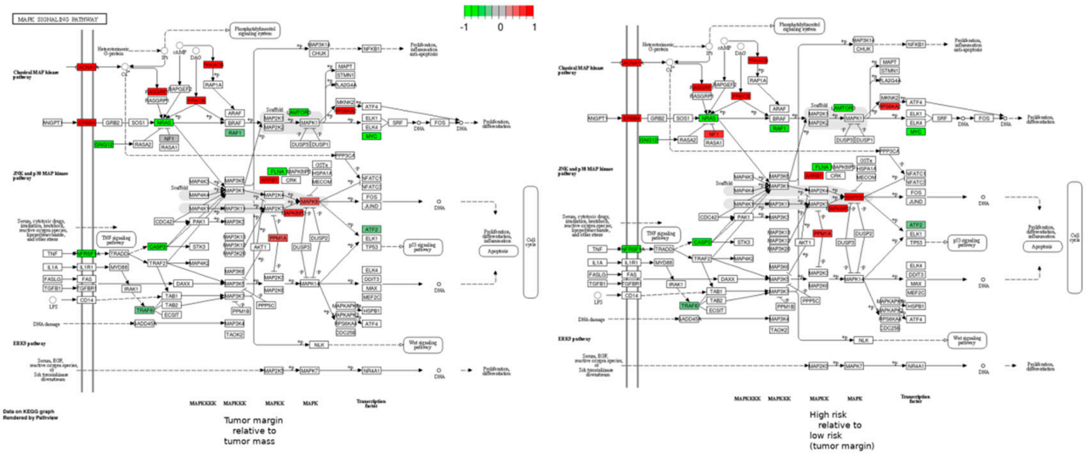

Figure S4. Potential recurrent-associated biomarkers in MAPK signaling pathway. CACNA1A, ERBB4, RASGRF1, PRKACB, NF1, RPS6KA2, ARRB1, MAPK9, MAPK8IP2, and PPM1A were identified in this signaling pathway.
